# Breaking Trivium Stream Cipher Implemented in ASIC Using Experimental Attacks and DFA

**DOI:** 10.3390/s20236909

**Published:** 2020-12-03

**Authors:** Francisco Eugenio Potestad-Ordóñez, Manuel Valencia-Barrero, Carmen Baena-Oliva, Pilar Parra-Fernández, Carlos Jesús Jiménez-Fernández

**Affiliations:** Departament of Electronic Technology, University of Seville, Virgen de Africa 7, 41011 Seville, Spain; manolov@us.es (M.V.-B.); cbaena@us.es (C.B.-O.); pparra@us.es (P.P.-F.); cjesus@us.es (C.J.J.-F.)

**Keywords:** fault attack, stream cipher, IoT, Trivium, ASIC implementation, DFA, experimental attack, key recovery

## Abstract

One of the best methods to improve the security of cryptographic systems used to exchange sensitive information is to attack them to find their vulnerabilities and to strengthen them in subsequent designs. Trivium stream cipher is one of the lightweight ciphers designed for security applications in the Internet of things (IoT). In this paper, we present a complete setup to attack ASIC implementations of Trivium which allows recovering the secret keys using the active non-invasive technique attack of clock manipulation, combined with Differential Fault Analysis (DFA) cryptanalysis. The attack system is able to inject effective transient faults into the Trivium in a clock cycle and sample the faulty output. Then, the internal state of the Trivium is recovered using the DFA cryptanalysis through the comparison between the correct and the faulty outputs. Finally, a backward version of Trivium was also designed to go back and get the secret keys from the initial internal states. The key recovery has been verified with numerous simulations data attacks and used with the experimental data obtained from the Application Specific Integrated Circuit (ASIC) Trivium. The secret key of the Trivium were recovered experimentally in 100% of the attempts, considering a real scenario and minimum assumptions.

## 1. Introduction

Electronic devices are nowadays characterized by the continuous exchange of information. The amount of information being exchanged is increasing day by day and even more with the development of the Internet of Things (IoT). Most of the information exchanged is sensitive, susceptible to be attacked by malicious agents. In [1,2] the authors pointed out the importance of security in the IoT and the challenges it poses, highlighting the need for secure implementations with severe constraints in terms of power consumption, area, and other features. A good example of the great interest and continuous progress in compromising the security of systems used in IoT applications is [3,4], where different ciphers are attacked.

Secure communications depend on the security of the used devices. In cryptography, two large groups of cryptographic algorithms are distinguished, those of public key (also known as ymmetric cryptography) and those of secret key (also called symmetric cryptography). In the first group the devices use two keys, one public and one private. The user encrypts the information with the public key and the recipient decrypts it with the private key. In contrast, in the second group the devices use only one key and this key is secret. This key must be the same when encrypting and decrypting the information and must be shared. Among the algorithms of public key cryptography, the Rivest, Shamir and Adleman (RSA) [5] and the algorithms based on Eliptic Curve Criptography (ECC) [6,7] stand out. Among the secret key algorithms, the Advanced Encryption Standard (AES) block cipher [8] is the standard approved by National Institute of Standards and Technology (NIST), but there are also many other proposals for lightweight applications such as LED [9] and stream ciphers such as Mickey [10] and Trivium [11].

However, there are continuous proposals of algorithms. Those that are linked to the NIST projects are highlighted. In the case of public key cryptography algorithms, there is a project for the standardization of public key cryptographic algorithms resistant to quantum computer attacks (post-quantum cryptography). In September 2020, the seven algorithms of the third round of candidates were made public [12]. For the secret key algorithms, NIST has opened another contest focused on secret key algorithms. This project is in its second round and there are 32 candidates [13].

On the other hand, there is also an effort in the efficient and secure hardware implementations of cryptographic algorithms. It should be noted that there are many works that study public key encryption implementations and analyze their robustness against different attacks. As a brief example for the reader, efficient implementations of public key algorithms (ECC and RSA) are presented in [14,15,16,17], and theoretical attacks are described in [18,19,20]. Due to the great number of encryption systems that exist, as mentioned above, at this point we focus on the analysis of private key ciphers because public key ciphers are out of our scope. Within the private key algorithms, there are many new cryptographic algorithms which attempt to balance security with severe constraints (lightweight cryptography) and are therefore constantly being developed. One of the most prominent options in lightweight cryptography are stream ciphers, which have simple structures and consume a limited amount of resources. An example of research and development on stream ciphers is the eSTREAM project [21]. Among the finalists, the Trivium stream cipher is one of the best options in terms of resources and power consumption and was selected as an ISO Standard (ISO/IEC 29192).

The security of cryptosystems is not easily threatened by attacks on the algorithm itself; however, it is possible to compromise their security by attacking their physical implementation, using for example side channel attacks or fault injections. Using fault injection mechanisms (laser beams, voltage peaks, clock glitches) or power consumption analysis, together with theoretical DFA [22] or Differential Power Analysis (DPA) [23], it is possible to cryptanalyze a cryptosystem and compromise its security. This type of cryptanalysis is based on a theoretical model that retrieves the secret information by comparing the correct output with faulty outputs.

The DFA technique combined with fault injections was used in [24] to cryptanalyze different cryptographic circuits, including a RSA. This work proved that this type of cryptographic circuits had vulnerabilities against this new analysis technique. On the other hand, the vulnerability of cryptographic systems such as RSA and DES, among others, was proved in [25] by using fault injection attacks combined with mathematical DFA analysis. Thanks to this work, the scientific community began to use fault analysis as a method to compromise the security of new cryptographic circuits.

They also led to the development of theoretical DFA based on transient fault injections. In [26,27,28,29,30] theoretical analyses of fault injections in different versions of AES are presented. This type of analysis has also been theoretically applied to a multitude of both block and stream ciphers, such as Midori, PRESENT, SIMON, SPECK, LED, MICKEY, GRAIN and PLANTLET [3,4,31,32,33,34,35,36,37]. All these works use the DFA method to theoretically recover the ciphers secret key, using transient fault injections and exploiting the obtained information. Of special interest for the present work, taking into account that the cipher under consideration is the Trivium stream cipher, are the theoretical DFA attacks reported in [38,39,40,41]. They are discussed in the next section in more detail.

**Figure 1:**
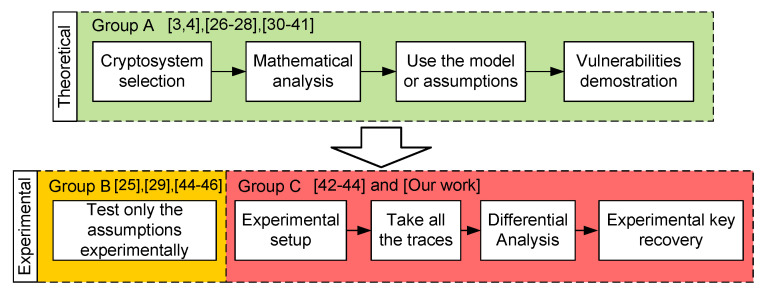
General classification of different type of cryptanalysis works.

A common feature to most of the DFA attacks presented in the literature is that the vulnerability of cryptosystems is theoretically studied. This is done by using attack models or by assuming a specific type of fault injection. A few of them have built an experimental setup to attack the system, sampling the outputs and performing the DFA. Depending on whether the cryptanalysis has been carried out theoretically or experimentally, we have classified the works into three groups, as it is shown in Figure 1. Group A includes works that use theoretical fault models or assumptions to prove the vulnerabilities of a cryptosystem. Group B includes works that in addition to the theoretical fault model, demonstrate the feasibility of the assumptions but do not attempt to built an experimental setup to recover the key. The last group, C, includes those works which present a complete setup and a description of the setup used to sample traces and experimentally recover the key in a hardware implementation.

All the works described in the preceding paragraphs belong to groups A and B because they assume that it is possible to achieve the theoretical assumptions and the fault model experimentally. This, however, is not necessary true because a fault model depends on many variables, such as inserting a specific number of faults or repeating the fault injection to insert faults in different positions. Reference [26] is a good example of this. It is a study with a good theoretical basis, but the authors of this paper claim that the theoretical fault models are very difficult to translate into a physical implementation. This difficulty makes it impossible to achieve the scenario theoretically proposed in the first model, and very difficult to do so in the second one. Another example is [30], where the authors present an experimental attack and prove that it is possible to inject faults, but later they conclude that the attacks must be carried out in a very specific scenario and they assume that the probabilities of achieving their models experimentally are very limited in other scenarios.

The number of studies within group C is small. One example of this kind of work is [42], where the key of an AES block cipher was recovered using a complete setup. They assume that it is very difficult to inject faults since, considering the large number of variables involved, the attack must be very finely adjusted. Another example is [43], where the authors retrieved the secret key of an AES using a complete experimental setup, taking into account the problems posed when theoretical assumptions are put into practice. In the case of the stream ciphers the number of works is even lower, being all of them presented using FPGAs implementations as [44] and as far as we know none of them applied to ASIC implementations.

In the case of the Trivium stream cipher the most important DFA analyses are the mentioned above [38,39,40,41]. These works are in Group A, and they base their analyses on ideal fault models or scenarios. None of them, however, prove whether it is possible to achieve assumptions experimentally. To correct this deficiency and to discover the weak points of the Trivium ciphers and improve their security, our paper presents a complete experimental setup and key recovery system. As far as we know, this complete setup (falling within Group C) is the first work in which it is experimentally proven that ASIC implementations of Trivium stream ciphers are vulnerable to real fault attacks and DFA by breaking its security.

### 1.1. Related Works

The DFA technique was used to theoretically demonstrate the vulnerability of the Trivium cipher in [38,39,40,41]. In these works, the simulation of fault injections in the cipher was carried out and the internal state information was obtained from the cipher faulty key streams. The main assumption to break its security is based on injecting a single faulty bit in the internal register of the cipher. Despite the theoretical study, none of these works prove the possibility of carrying out such attacks in the real world with physical implementations of the cryptographic circuit. This aspect is covered in detail in Section 2 of this paper, which also explains the number of injections needed to retrieve the key using DFA.

Experimental attacks on the cipher were presented in [45,46,47] (Group B), where different attack systems were designed to achieve the assumptions of theoretical attacks. These studies, carried out on FPGA implementations, gave rise to positive results for the injection of effective faults, with a high percentage of effectiveness and efficiency. However, none of these works attempted to built the setup that would make it possible to retrieve the secret key experimentally in a real world scenario, where the attack is performed externally to the implementation and where multiple variables must be taken into account.

### 1.2. Our Contribution

This paper presents the first complete setup to experimentally retrieve the secret key of the Trivium cipher, combining a theoretical DFA and the experimental results of attacks on ASIC implementations. A complete study was made, using simulation attacks to determine the number of injected faults necessary to recover the internal state of the cipher. In addition, a study of the number of faults injected as a function of the fault frequency used in different clock cycles was made. The theoretical DFA presented by Michal Hojsïk and Bohuslav Rudolf [38,39], was modified and adapted to be included, for the first time, in a complete attack system that is able to exploit the results obtained experimentally from an ASIC. Due to the difficulty of injecting faults in different positions of the Trivium internal state in a non-invasive way and in the same clock cycle, it has taken advantage of the fact that stream ciphers base their structures on shift registers. We also present a backward design of Trivium which allows the recovery of the secret key from any internal state retrieved in any clock cycle. The combination of DFA and experimental results made it possible to fully recover the secret keys in 100% of cases (both experimentally and in simulation). The keys were recovered in two different ASIC implementations of the Trivium cipher, taking only few minutes.

### 1.3. Paper Organization

The rest of the paper is organized as follows. Section 2 presents a brief description of the Trivium stream cipher architecture and its characteristics, and the main features of the DFA for a Trivium cipher, focusing on the main assumptions taken into account in order to retrieve the secret key. Section 3 presents the setup of the DFA software to obtain the internal state. Modifications to the original DFA software are explained, describing the simulated fault attack carried out to test the possible combination of DFA and experimental fault attacks. In addition, some results of the functionality check are shown. Section 4 presents the attack planning, describing the cipher implemented on ASIC, the attack method, the challenges of the fault frequencies and the ciphers vulnerabilities. Section 5 describes how to achieve multiple faults in the Trivium cipher, and the design of a backward Trivium that allows obtaining the secret key. Section 6 presents the results obtained from the experimental attacks on the two ASIC implementations of the Trivium cipher and explains how the key was recovered. Finally, some conclusions are given in Section 7.

## 2. Differential Fault Analysis for Trivium

### 2.1. Description of the Trivium Stream Cipher

**Figure 2:**
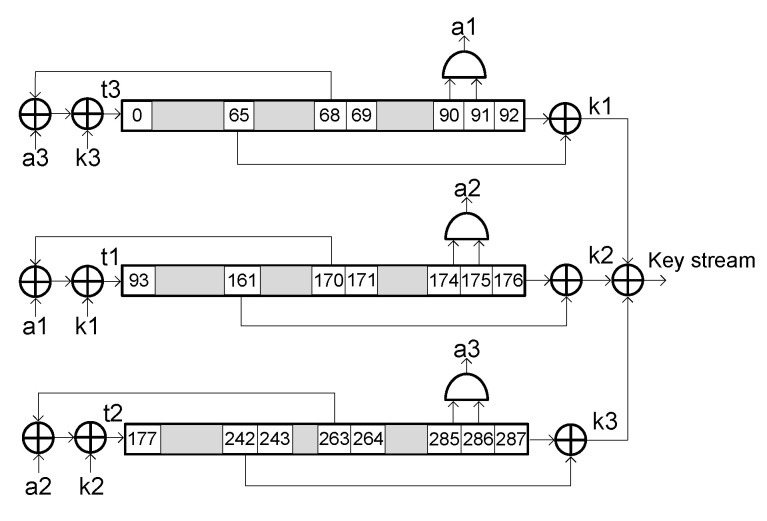
Schematic representation of the Trivium stream cipher internal structure.

The Trivium stream cipher [11] was one of the eSTREAM project finalists along with the MICKEY and GRAIN ciphers, and is part of the ISO/IEC 29192-3 standard for lightweight stream ciphers. From an 80-bit secret key denoted as KEY and an 80-bit initialization vector denoted as IV, this cipher is able to generate in a synchronous way up to $2^{64}$ bits of key stream. Figure 2 shows a schematic representation of Trivium internal structure. As it can be seen, its internal structure is performed by three shift registers comprising 288 bits in total and ten XOR gates and three AND gates for the feedbacks. Each of these three shift registers are composed by 93, 84 and 111 bits respectively. The KEY and the IV are loaded in the internal register, along with some prefixed zeros and ones. After the first 1152 clock cycles, the cipher generates a valid pseudorandom bit sequence. The key stream is the result of the XOR operations.

### 2.2. DFA on Trivium

DFA is essentially a theoretical attack where if any attacker is able to inject transient faults into the operation of a device (either in its encryption or decryption processes) through the use of mathematical formulation, can obtain secret information contained in the device and thus endanger its security. Of the different assumptions necessary to carry out DFA on the Trivium stream cipher, the most important one is that the attacker is able to inject a single effective fault into the ciphers internal state and capture both the correct key stream and the one originated by that fault. For our system it is necessary to capture 800 bits. A more complete description of the mathematical aspects of the DFA system can be found in the references [38,39].

In summary, and taking into account DFA nomenclature, the attacker is able to obtain both the key stream of the Trivium cipher ${\left\{z_{i}\right\}}_{i=1}^{\infty }$ produced by its internal state $ISt_{0}$ and the key stream produced from a transient random fault ${\left\{z_{i}^{\prime }\right\}}_{i=1}^{\infty }$, introduced into the internal state which is now called $IS^{\prime }t_{0}$ because it contains the fault.

The same attack and the capture of the faulty key stream must be carried out repeatedly under the same conditions: i.e., using an ISt loaded with the same key and IV and always attacking in the same clock cycle $t_{0}$. With the system designed by Hojsík and Rudolf, it is possible to analyze the differences between the correct key streams and the faulty key stream produced by the fault injections and to determine the values of the ciphers internal state 800 clock cycles after the attack clock cycle (t=1155+X+800).

### 2.3. Attack Assumptions

At this point, let us clarify the difference between theoretical and practical assumptions. On the one hand are the theoretical assumptions without which it is not possible to recover the cipher key by means of DFA and on the other hand, the practical assumptions, which are those taken by a real attacker. The theoretical assumptions are:

1. The time of the attack, $t_{0}$ (clock cycle) must be a fixed positive integer and the internal state of the cipher, $ISt_{0}$, it is unknown but fixed. The clock cycle is selected after the 1152 configuration clock cycles.
2. A single fault can be inserted in the internal state of the cipher in an unknown position: i.e., a single bit can be changed to an incorrect value, either 0 to 1 or 1 to 0.
3. After $t_{0}$, *n* bits of the correct key stream (produced by internal register $ISt_{0}$, without the fault injected) and the faulty key stream (produced by internal register $IS^{\prime }t_{0}$, with the fault injected) can be captured.
4. The attack process (restart, insert fault and capture the wrong key stream) can be performed several *m* times.
5. Faults must be injected in different positions of the internal state by repeating the attacks in the same clock cycle $t_{0}$.

**Figure 3:**
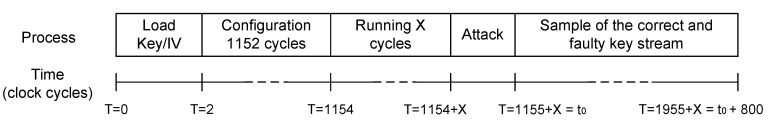
Representation of the attack and acquisition process.

This process is depicted graphically in Figure 3. which outlines the loading, operation, attack, acquisition and processing sequence. To carry out the DFA, the most important consideration is that it is necessary to insert a single fault in the internal state register. The value of only one flip-flop can be changed. Each fault must also be injected at a different position.

In the case of practical assumptions we have considered the minimum requirements necessary to perform an attack and endanger the security of any cryptocircuit in real scenario. The practical assumptions are those where the attacker:

1. Has access to a device with the Trivium cipher implemented.
2. Can restart (reset) the system as many times as he needs, with the same -although unknown- secret key and IV.
3. Has access to the cryptographic device output key stream: i.e., can capture the device key stream output.

To meet the theoretical assumptions, if the attacker has access to a device with the cipher (in our case an ASIC), the attacker can control the reset and the clock and is able to sample the key stream from the output of the circuit (all of them are input and output signals). Therefore, the attacker is able to select the attack clock cycle, reset the cipher many times and to know if the cipher is working properly or not by observing the output. The attacker needs to count the clock cycles of the circuit and attack it at a given time (theoretically set as $t_{0}$) and then observe the output. The attacker does not know if the result of the attack is satisfactory or not, because it is not possible to know if a single or several faults have been injected. The DFA tool must generate different messages if the injected fault is not an effective fault. Please note that these last three assumptions are the minimum and necessary if an active, non-invasive attack is to be carried out, which is necessary for a DFA analysis. Otherwise, DFA would not be applicable.

## 3. Set Up of the DFA System to Retrieve the Internal State

To break the Trivium stream cipher, we took into account the theoretical works of Michal and Bohuslav presented in [38,39]. Besides being the first works to carry out DFAs on the Trivium cipher, these studies describe a hypothetical attack scenario and the assumptions that must be taken into account to carry out the attack. Please note that this analysis is always performed in an ideal scenario where external variables or synchronization problems are not considered.

The goal of the DFA system developed by Michal and Bohuslav was to theoretically demonstrate the vulnerability of the Trivium cipher to DFA attacks. In their software they internally emulate the operation of a Trivium cipher initialized with a random key and IV, insert a random fault and generate the 800 bits of the correct and faulty key streams. They introduce the data of these key streams into a system of linear equations with variables associated with each of the three shift registers of the Trivium. Solving the equation system, the software determines the number of bits discovered in the internal register of the cipher. If the number of faults and the positions at which they were inserted were adequate and enough, all 288 bits can be discovered. In the cases where there were not enough faults, it can only determine some of them. The original software therefore returned:

1. The number of bits from the internal state discovered (out of the possible 288), but not their value or position in the internal state.
2. An error when, having reached a maximum number of injected faults, it had not been possible to obtain the 288 bits of the internal state.

### 3.1. Modifications to the Original DFA System

Our priorities were, firstly, to merge the DFA software into the experimental setup and use data obtained experimentally and, secondly, to obtain the key and the IV used for the encryption. To achieve these objectives, the following modifications and additions were made to the original software:

1. External data reading. The key stream data is read from files containing experimentally captured data. Each file has a line of 800 characters representing the 800 bits of the captured key stream. One of the files contains the correct key stream and the rest contain faulty key streams generated from the injected faults.
2. Display the bits recovered with the injected faults. With this modification, the user is notified of the number of bits of the internal state recovered, according to the number of injected faults.
3. Representation of the internal state values in binary and hexadecimal format. The original system calculated the number of recovered positions of the internal state but showed neither their positions nor their values. Our modification made it possible to extract the recovered bits of the internal state (position and binary value), and write them in a file, for the clock cycle $t_{0}+800$.

With these modifications, a DFA system that can exploit the results from experimental and simulation fault injections was designed for the first time. The new system is able to return the ciphers internal state value at clock cycle $t_{0}+800$.

### 3.2. Simulation Verification of the New DFA System

**Figure 4:**
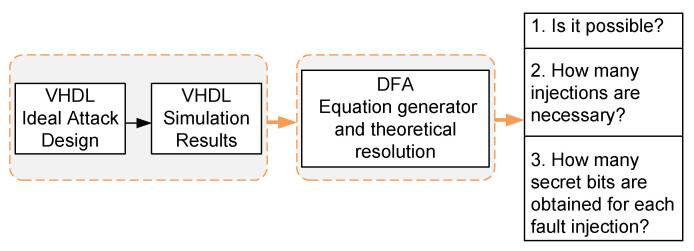
Representation of the combination of the two main blocks.

To verify the correct operation of the developed DFA system, it was first tested with data from simulations. The ISE 14.7 tools of Xilinx and Eclipse have been used to carry out the simulations. To simulate in VHDL ideally the fault injections the ISE functional simulations designed in VHDL have been used and for the analysis of the faulty key streams the Eclipse tool with C code for DFA has been used. The simulation was done by creating a VHDL design that could introduce a fault in the internal state of a Trivium cipher and capture the 800 bits of the key stream. This design will also help us to identify the requirements for the experimental attack, such as the number of bits of the internal state recovered according to the number of faults introduced and the influence of the clock cycle of the attack. A block diagram of the verification system can be seen in Figure 4. It shows how the two main blocks –the simulation attack system and the DFA– are interconnected. The simulation injection system has the following characteristics:

1. Random selection of secret key and IV.
2. Selection of the clock cycle of the fault injection.
3. Selection of the position of the internal state where the fault is injected.
4. Acquisition of the key stream produced by the cipher.
5. Copy of the key streams to a file for later use in the DFA system.

The simulation tests verified the correct operation of the DFA system. The 288 bits of the internal state were obtained regardless of the key and IV used and also the clock cycle in which the faults were injected. In Algorithm 1 the process of the ideal attack carried out on the t1 feedback is described. This process is equally applicable to both t2 and t3 but for simplicity is only described the process for t1. Please note that the ideal attack in VHDL is equivalent to Figure 3 since it graphically describes the ideal attack and capture process of the key stream.
**Algorithm 1:** Ideal Attack Process—Fault Injection.**Require:** Attack Clock Cycle (ACC)**Require:** Fault = 1 **return** Key stream ${\left\{z_{i}\right\}}_{i=1}^{N}$ **for** i={1}to{N} **do**
  $z_{i}\leftarrow S_{65}⨁S_{92}⨁S_{161}⨁S_{176}⨁S_{242}⨁S_{287}$  **if** i=ACC **then**
   $t_{1}\leftarrow S_{65}⨁S_{90}•S_{91}⨁S_{92}⨁S_{170}⨁Fault$  **else**
   $t_{1}\leftarrow S_{65}⨁S_{90}•S_{91}⨁S_{92}⨁S_{170}$  **end if**  $t_{2}\leftarrow S_{161}⨁S_{174}•S_{175}⨁S_{176}⨁S_{263}$  $t_{3}\leftarrow S_{242}⨁S_{285}•S_{286}⨁S_{287}⨁S_{68}$  $\left(S_{0},S_{1},\ldots ,S_{92}\right)\leftarrow \left(t_{3},S_{0},\ldots ,S_{91}\right)$  $\left(S_{93},S_{94},\ldots ,S_{176}\right)\leftarrow \left(t_{1},S_{93},\ldots ,S_{175}\right)$  $\left(S_{177},S_{178},\ldots ,S_{287}\right)\leftarrow \left(t_{2},S_{177},\ldots ,S_{286}\right)$ **end for**


### 3.3. Analysis by Simulation

Once the correct operation of the DFA system had been checked, the simulation data was used to know how many bits of the internal register are obtained as a function of the number of fault injections carried out, and how many fault injections are necessary to recover the 288 bits of the internal state. It was also interesting to know whether the positions of the injected faults affect the number of internal state bits recovered.

Table 1 shows the number of internal state bits recovered for different numbers of fault injections for five randomly chosen key and IV values (Cases 1 to 5).

The number of fault injections required to retrieve all the bits of the internal state ranged from 16 (Case 1) to 24 (Case 5). However, it can be seen that, in all cases, with 16 fault injections the number of recovered bits was very close to the total number of bits of the internal state. The worst case is Case 5 in which, with 16 fault injections, 6 bits of the internal state remained unrecovered, but those 6 bits can be easily recovered by brute force. Many more cases were tested and their results fell within the range of those shown in Table 1.

Finally, we studied the influence of the order of the fault injection positions on the number of bits recovered from the internal state. Table 2 shows the bits recovered after injecting the faults in the same three positions (1, 2 and 3) but in all possible order combinations when the DFA is applied. As it can be seen in the table, if the order of the fault injections is modified, the same number of bits -in this case 88- are always recovered. This study was carried out using the same key and IV.

From the results obtained through simulation, it can be concluded that the values of the key and the IV have very small effect on the number of fault injections needed to recover the internal state of the Trivium. For the same key and IV, the order of the injected faults does not affect the DFA.

## 4. Attack Planning

### 4.1. Trivium Implemented on ASIC

The experimental attacks were carried out in two Trivium ciphers implemented in a 90 nm ASIC technology. The key and IV to be used are loaded serially in the ASIC, and the clock and control signals of the Triviums are connected to the ASIC input pads. The key streams of the Triviums are connected to the output pads of the ASIC.

The attack described here was performed on two copies of the Trivium cipher that share the same input signals (clock and control signals), and with the same key and the same IV. The only difference is the routing of the two Triviums.

### 4.2. Attack Using Clock Glitches

Fault attacks can be performed with different techniques. Laser beam or electromagnetic attacks allow great control over the inserted faults, but they need an invasive manipulation of circuits, such as stripping. In addition, these techniques are quite expensive and can cause permanent damage to the circuit. Injecting faults in ciphers such as Trivium is not an easy task, since the cipher structure is based on shift registers, and they can operate at very high frequencies, being close or above to the maximum operating frequencies supported by the technology. To inject the faults into the cipher an active non-invasive fault injection system has been designed. It is based on inserting short pulses in the clock signal. This technique allows violating the setup times of the flip-flops making the sampled value on its output be erroneous which represents a fault injection in the Trivium cipher. This technique has proven to be efficient when applied to FPGA implementations of the Trivium cipher [45,46,47], but it has not been tested in ASIC implementations. In [45,46,47] the clock signal was internally generated using Digital Clock Managers (DCMs), however, the attack over the circuit presented in this paper is externally performed. The clock signal with the short pulses is externally generated and has to pass through the input pads of the integrated circuit to reach the circuit. This is a great challenge because low frequencies will not inject faults in the circuit, but very high frequencies can be filtered by the circuit pads. The search for a proper way to introduce short pulses into the clock signal not filtered by the pads and that could introduce effective faults is therefore a big challenge that we have to face. The fault injection system presented can be applied also to others ciphers implemented on ASIC [48,49], FPGAs [50,51,52] or microcontrollers [53,54]. In this case, the signals should be modified to adapt the inputs and outputs to match the target circuit.

The Triviums implemented in the ASIC have been attacked using the Agilent 93,000 test system. Agilent 93,000 allows generating and changing the signals very accurately, and to sample the output data. It also allows generating short pulses in the clock signal at the desired clock cycle. One of the main advantages of this tool is the possibility to control in a very precise manner the time delays between the different signals. In addition, this tool allows avoiding any possible uncertainty in terms of time delays or sampling errors. It should be notice that we have used this test system because we have access to it, but the attack is also feasible with cheaper test systems. In order to carry out successful attacks, the attacker must have an accurate control of the clock signals and delays.

**Figure 5:**
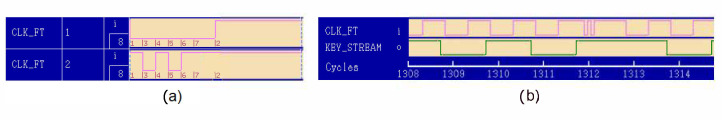
Screen capture of: (**a**) waveforms to be applied, (**b**) clock signal with the short pulse.

The generation and insertion of the short pulse in the clock signal is done through the definition of different waveforms for the same signal. Agilent 93,000 allows the definition of these waveforms and their use in the selected clock cycles. Figure 5 shows two screenshots of the window used to configure the generation of the clock and the short pulse. On Figure 5a it is shown the two different waveforms defined for clock signal (CLK_FT). The top waveform (waveform 1) is used for the normal clock signal and the bottom waveform (waveform 2) is used to insert a short pulse. The short clock pulse has a higher frequency than the maximum operating frequency of the Trivium cipher. This allows injecting faults in the internal register composed by flip-flops. The clock signal is generated by selecting the waveform to be used in each cycle as it is shown in Figure 5b. In this case waveform 1 is used in all cycles except in the cycle 1312 where waveform 2 is used and one short pulse is generated. The sampled key stream is also showed in Figure 5b.

With the mechanism for generating and inserting pulses into the clock signal, the first step is to experimentally analyze the number of faults injected into the internal state as a function of the fault frequency used for the pulses. Table 3 shows the number of faults injected in the cipher internal state for different clock cycles and fault frequencies. When the number is zero it means that no error has been injected. If the number is one it means that the attack has been satisfactory and a single fault has been injected. When the value is greater than one it means that multiple errors have been injected. These data were obtained by sampling the internal register of the cipher. The results shows that the attack system is able to inject effective faults into the cipher. As it can be seen, the difference between the frequency that produces multiple faults and the frequency that does not produce any fault is very small. We need frequencies between 143 MHz and 144 MHz to inject faults in the ciphers. These frequencies allow inject effective faults, but it can inject multiple faults also. In the case of that multiple faults where injected, the DFA system will produce an error. A frequency of 25 MHz has been used as the main clock signal.

### 4.3. Trivium Vulnerabilities

The results presented in previous works [45,46,47] for attacks carried out by manipulating the clock signal in FPGA implementations of the Trivium showed that it is possible to inject faults only in flip-flops with feedback inputs: namely, position bits 0, 93 and 177 of the internal register or its neighbouring flip-flops. In addition, even with these flip-flops, faults can only be introduced in those that change their value. It is therefore only possible to obtain an average of three faulty key streams. Tests carried out on ASIC implementations showed the same behaviour: faults are injected into flip-flops whose inputs come from feedback or their neighbours.

This is a serious problem because, as explained in Section 4, in order to recover the internal state of the Trivium using the developed DFA, it is necessary to have between 16 and 24 faulty key streams. Therefore, the developed DFA system requires faulty key streams generated by faults injected in at least 16 different positions for the same clock cycle. The fewer faulty key streams we have, the greater the brute force effort needed to recover the internal state of the cipher.

With only three faulty key streams it is not possible to recover the internal state. Therefore, in order to successfully use the developed DFA system, it was necessary to modify the fault injection mechanism to be able to inject the faults in more positions in the internal register of the ciphers. The following section explains how to achieve this.

## 5. Recovery of the Secret Key Experimentally

### 5.1. Achieving Multiple Faults in the Same Clock Cycle

It is possible to inject faults into more internal flip-flops because stream ciphers in general and Trivium in particular are built with shift registers. The shift registers make the fault injected into a flip-flop in one clock cycle appear as a fault injected in the next position of the shift register in the next clock cycle. This technique was presented mathematically in [44] for the Grain stream cipher and we have adapted it for the Trivium cipher. This section explains in detail the fault injection strategy applied to the Trivium cipher, which makes it possible to successfully obtain the number of faults needed to carry out the experimental DFA.

To understand how this fault injection strategy works in a Trivium stream cipher, it is necessary to consider the design of the cipher internal registers. As it can be seen from the schematic representation of the Trivium cipher shown in Figure 2, Trivium is based on three shift registers with non-linear feedbacks. In these shift registers, the first flip-flops used for the feedbacks and to generate the key stream are the flip-flops in positions 65, 161, and 242.

**Figure 6:**
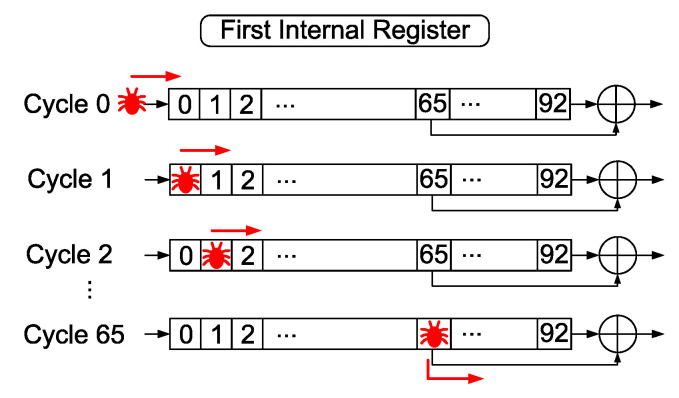
Propagation of the injected fault through the first register.

If a fault is injected into the first bit of any of the three shift registers (0, 93, 177), the faulty bit will not contribute to the key stream generation until it reaches one of the bits used for key stream generation (65, 161, and 242). This behavior can be seen schematically in Figure 6 for the first shift register, where the fault does not affect the key stream until it reaches the 65th position of the register. During these clock cycles, the fault is only shifted through the shift register. Inject a fault in position 0, is therefore equivalent to introduce one fault in the position 0+n, *n* clock cycles later. This strategy can greatly increase the number of positions in which faults can be injected.

For example, if cycle $t_{0}=1325$ is set as the attack cycle for DFA, and a fault is injected at position 0 of the internal register 25 cycles earlier, i.e., at $t_{-25}=1300$, the fault will move along the register in each clock cycle. When 25 clock cycles have passed, we are in the cycle established for the differential analysis ($t_{0}=1325$), and the fault is at position 25 of the state register. Therefore, the behaviour is the same as if we had injected a fault in the flip-flop 25 of the state register.

Applying this method to the Trivium, it is therefore possible to inject 65 faults (from bit 0 to bit 65) in the first shift register, 68 faults (from bit 93 to bit 161) in the second shift register and 65 faults (from bit 177 to bit 242) in the third shift register.

A fault attack would be as follows:

1. Cycle 1365 is set as the attack clock cycle ($t_{0}$).
2. The number of attacks needed is set: for example, 65 attacks.
3. The first attack is carried out in cycle $t_{-65}=1300$.
4. The second attack is carried out in cycle $t_{-64}=1301$.
5. This process is repeated 65 times, $t_{-63}=1302$, $t_{-62}=1303$, etc.
6. When cycle $t_{0}=1365$ is reached, 65 error injections have been carried out and 65 faulty key streams have been obtained.

Please note that to carry out the DFA successfully, it is necessary to obtain 800 bits of the key stream, always from the clock cycle $t_{0}$ selected for the attack. The sampling of the key stream starts once the selected clock cycle for the attack has been reached.

Extending this analysis to all three shift registers, it is possible to inject faults in the same clock cycle in the following ranges of positions:

1. For the first shift register it is possible to insert faults from position 0 to position 65.
2. For the second shift register it is possible to insert faults from position 93 to position 161.
3. For the third shift register it is possible to insert faults from position 177 to position 242.

This attack strategy, applied in the experimental setup developed, allows injecting faults in many different positions, easily surpassing the requirements presented in Section 3 (at least 16 fault injections in 16 different positions).

### 5.2. Complete Attack and Backward Trivium

Once we have the complete setup and the strategy to obtain the faulty key streams needed for the DFA system, the next step is to apply the whole experimental cryptanalysis system and recover the secret key from a real Trivium cipher implemented in an ASIC. For this cryptanalysis process, it was necessary to develop a general attack plan consisting of:

1. Configuring the ciphers implemented in ASIC: random key and IV.
2. Selecting the attack clock cycle $t_{0}$.
3. Carrying out successive attacks with the test system and sampling non-faulty and faulty key streams.
4. Applying the DFA.
5. Obtaining the internal register of the cipher at $t_{800}=800+1155+X$.
6. Run the Trivium in reverse to find out the internal state of the Trivium in the initial clock cycle (t=0).
7. Retrieving the secret key and IV.

**Figure 7:**
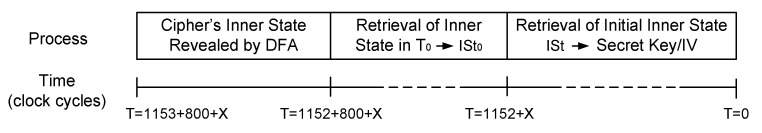
Representation of the secret key and IV recovery process.

Steps 1 to 5 correspond to the process shown in Figure 3, while steps 6 and 7 corresponds to the process shown in Figure 7. This attack plan is feasible in a real scenario, where an attacker has access to the cryptographic device and can manipulate the input signals and read the outputs.

**Figure 8:**
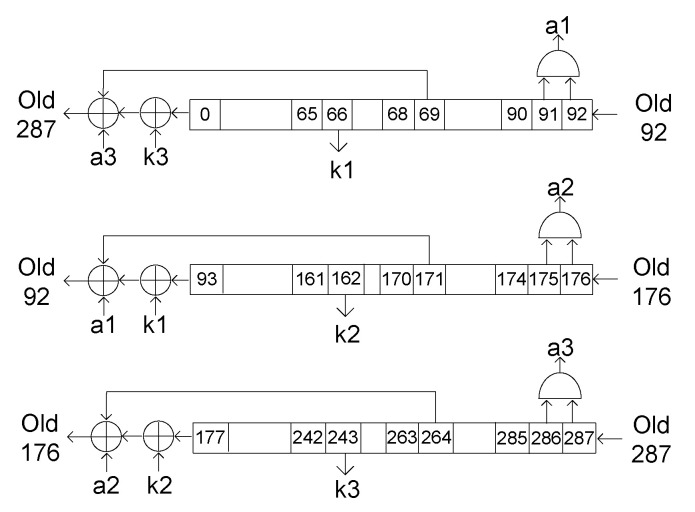
Schematic representation of the backward Trivium design.

To recover the internal state at the initial time, an inverse-operation Trivium cipher was designed. The pseudo-code of this cipher, represented schematically in Figure 8, is shown in Algorithm 2. The Trivium uses some bits of the state register to generate the feedback and then shifted them one position each clock cycle. If we take the bits shifted by one position and carry out the operations in reverse, the result is the previous state. By repeating this process as many cycles as necessary, it is possible to obtain the initial internal state.
**Algorithm 2:** Backward Trivium.**Require:** Number of clock cycles $n=1153+X+t_{800}$. **for** i={1} **do**
  **if** $S_{93}=S_{66}⨁S_{171}⨁S_{91}\cdot S_{92}$ **then**
   $S_{92}^{\prime }\leftarrow 1$ **else** $S_{92}^{\prime }\leftarrow 0$  **end if**  **if** $S_{177}=S_{162}⨁S_{264}⨁S_{175}\cdot S_{176}$ **then**
   $S_{176}^{\prime }\leftarrow 1$ **else** $S_{176}^{\prime }\leftarrow 0$  **end if**  **if** $S_{0}=S_{243}⨁S_{69}⨁S_{286}\cdot S_{287}$ **then**
   $S_{287}^{\prime }\leftarrow 1$ **else** $S_{287}^{\prime }\leftarrow 0$  **end if**  $\left(S_{0},S_{1},\ldots ,S_{92}\right)\leftarrow \left(S_{1},S_{92},\ldots ,S_{92}^{\prime }\right)$  $\left(S_{93},S_{94},\ldots ,S_{176}\right)\leftarrow \left(S_{94},S_{176},\ldots ,S_{176}^{\prime }\right)$  $\left(S_{177},S_{178},\ldots ,S_{287}\right)\leftarrow \left(S_{178},S_{287},\ldots ,S_{287}^{\prime }\right)$ **end for**

For example, in the first shift register (see Figure 2), bits 90 and 91 were used to generate value “a1”. Once the internal register was known, at any time, it is possible to know the previous value of “a1” using bits 91 and 92, as shown in Figure 8. With this, all the values of the feedback were revealed and it is possible to operate the cipher backwards. With this design, knowing the state register of the Trivium in a clock cycle, it is possible to know the state register in any previous cycle.

After loading the secret key, the initial state consists of 288 bits with the following distribution:

$\left(S_{0},S_{1},\ldots ,S_{92}\right)\leftarrow \left(k_{0},k_{1},\ldots ,k_{79},0,\ldots ,0\right)$
$\left(S_{93},S_{94},\ldots ,S_{176}\right)\leftarrow \left(iv_{0},iv_{1},\ldots ,iv_{79},0,\ldots ,0\right)$
$\left(S_{177},S_{178},\ldots ,S_{287}\right)\leftarrow \left(0,\ldots ,0,1,1,1\right)$

Therefore, to calculate the key and the IV it is not necessary to know the number of clock cycles that have elapsed from the loading of the key and the IV to the injection of faults. When the Trivium reaches a content of the internal register as shown in the previous equations, the key and the IV have been obtained, and also the number of cycles that have elapsed.

## 6. Results

**Figure 9:**
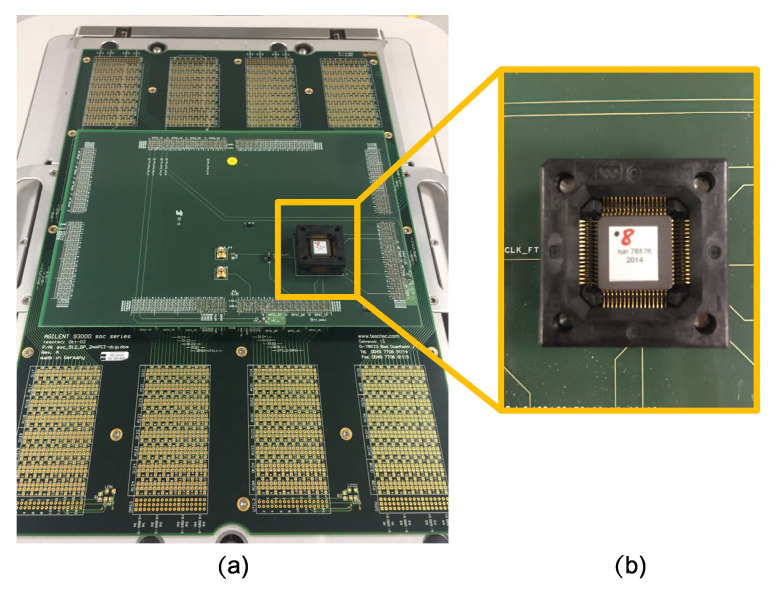
Experimental setup. (**a**) the ASIC test board. (**b**) one of the ASICs used to carry out the attacks on Trivium ciphers.

In this section we present the results obtained from the attacks carried out on two Trivium cipher implemented in an ASIC. The ASIC technology is TSMC 90 nm and the total area of the die is 1875 × 1875 μ m. The package is a CQFP of 64 pins. In addition, a specific Device Interface Board has been designed to make the electrical connections between the device under test and the test equipment. The only difference between the two ciphers is the routing in the ASIC. The results were obtained using the Agilent 93000 test system for the fault injection attacks, Eclipse Software for the DFA software, and Matlab for the Trivium backward cipher. The computer used to run the DFA and the backward Trivium was a Core-i5 desktop PC with 8 GB of RAM. The experimental setup is shown in Figure 9, with (a) the Agilent 93000 test board and (b) an image of the ASIC with the Triviums.

To carry out the attack on the two Triviums, we have used random keys and IVs. The attack cycle was set to $t_{0}=1332$, and the succession of attacks started in cycle $t_{-20}=1312$. Table 4 shows the results of the attack for Trivium 1 and Table 5 the results for Trivium 2. Each table includes the number of the fault injection attempt, the fault injection cycle, the relative position of the injected fault (as if it was a fault inserted in cycle $t_{0}=1332$) and the number of bits of internal state retrieved by the DFA.

The relative position of the injected fault corresponds to the position of the fault referenced to cycle $t_{0}=1332$. For example, in Table 4, Injection 1, cycle 1312, the fault was injected in position 94. Taking into account that the attack was made in cycle $t_{-20}=1312$, at the attack cycle $t_{0}=1332$ (21 clock cycles later), the fault was moved to the position 94 + 21 = 115.

The total number of fault injections attempts was 32. When “−” appears in the column “number of bits retrieved”, it means that the number of bits retrieved has not increased. Please note that the higher the number of injections, the lower the number of new bits retrieved. As already explained, faults are not always injected in the feedback positions. In some cases, faults are injected in adjacent positions. In these cases the relative position of the fault usually coincides with the position of the previous attack, and therefore the same faulty key stream is generated. This means that only one of the two attempts can be used to find new bits of the internal register. Taking this into account, although the number of fault injections attempts was 32, the number of injections used to perform the DFA was 22, corresponding to the non-repeating values.

On the other hand, and as it is shown in both tables for the two Triviums, the positions of the injected faults and the number of bits retrieved are different. This demonstrates a small difference, due to routing, in the behavior of the Trivium against this type of attack in ASIC implementations. For the same attack, under the same conditions, the two ciphers have different fault injection positions. However, in both cases the 288 bits of the cipher internal states are recovered and the secret key can therefore be obtained. We have repeated the attacks using different, randomly chosen, keys and IV, obtaining the secret key in 100% of cases.

The time to recover the secret key experimentally was about 6 h. Almost all of this time was spent on cipher fault injection and data capture. The attack process (fault injection and capture) was carried out one by one. This process involves selecting the fault insertion clock cycle, system operation, fault insertion, data capture and analysis of the result to find out if the inserted fault is useful or not. As it has been seen in the results, not always an inserted fault is useful, being necessary to select another attack clock cycle and repeat the process. Of course this process can be automated and therefore the cost in time would be much lower, but in this case as it is a proof of concept it has not been carried out. On the other hand, the processing time or the computational complexity or our solution is minimum. The time needed to process the data with a Core-i5 and 8 GB RAM PC is less than 20 s. Therefore, if the fault injection process was automated, it would be possible to recover the key of the Trivium cipher in just a few minutes.

Finally, the designed attack system is also applicable to other stream ciphers such as Mickey and Grain. These ciphers have been analyzed using DFA and the assumptions are similar to those of the Trivium cipher, i.e., injecting faults into their registers and capturing their faulty key streams in order to analyze them [35,36]. Therefore, it must be taken into account that since each of these ciphers has its own DFA analysis, in order to apply our experimental key recovery and attack system, the part related to the specific DFA analysis would have to be modified for each one and the attack process could be carried out in the same way as with the Trivium cipher.

## 7. Conclusions

This work describes the complete experimental breaking of Trivium ciphers implemented in ASIC technology. In 100% of the attempts, the secret key and IV were retrieved with minimal assumptions and in a real scenario.

The main milestones were: Firstly, experimental attacks were performed injecting a single fault into the internal register of the Trivium ciphers changing the external clock signal. Secondly, to inject faults in many positions of the internal register, we took advantage of the fact that the Trivium is built on the basis of shift registers. This has made it possible to obtain a sufficient number of faulty key streams to recover all the bits of the internal state of the cipher. Thirdly, an inverse-operation Trivium was designed to get the secret key from a known internal state. The achievement of these three steps, together with the developed setup has allowed to obtain the secret key of the Trivium implemented in the ASIC.

In addition, the correct operation of the system has been verified not only by the knowledge of the key used in the cipher, but also by the use of data generated by simulation. Using the data generated by simulation it was possible to know that to obtain the secret key with the developed system it is necessary to inject faults in at least 16 different positions of the internal state of the Trivium. For the experimental attacks that we have carried out, we have used 32 fault injection attempts. We have discarded those attempts that do not introduce faults in new positions, being able to obtain the secret key of the Trivium in all the attempted cases. It should be noted that the experimental attack system we have presented is also applicable to other stream ciphers. The only part that should be modified is the mathematical analysis to obtain the internal state from the obtained key streams, since this part is specific to each cipher.

In conclusion, the work we have presented demonstrates that it is possible to experimentally break the security of ASIC implementations of the Trivium cipher using fault attacks and Differential Fault Analysis, in a short time and in a real scenario.

## Figures and Tables

**Table 1:** Number of injections needed to obtain 288 bits of the internal state.

Num. Fault Injections	1	4	8	12	16	17	18	19	20	24
Case 1	21	99	195	272	288	–	–	–	–	–
Case 2	20	92	190	272	285	288	–	–	–	–
Case 3	21	89	252	278	284	287	288	–	–	–
Case 4	28	105	181	260	284	284	285	287	288	–
Case 5	28	100	189	238	282	283	285	285	287	288

**Table 2:** Number of internal state bits revealed in function of the fault position used in DFA.

Fault 1	25		Fault 1	25		Fault 2	36		Fault 2	36		Fault 3	25		Fault 3	25
Fault 2	62		Fault 3	51		Fault 1	62		Fault 3	61		Fault 1	51		Fault 2	61
Fault 3	88		Fault 2	88		Fault 3	88		Fault 1	88		Fault 2	88		Fault 1	88

**Table 3:** Number of fault injected in the cipher per clock cycle and fault frequency.

	Fault Frequency (MHz)				
Clock Cycles	142	142.2	143	143.88	150
1307	0	0	127	127	127
1312	0	1	2	2	60
1402	0	0	0	138	122
1403	0	0	0	1	138
1545	0	1	1	1	60
1546	0	0	0	142	142

**Table 4:** Results obtained from the attack on Trivium 1.

F.I.A. ${}^{1}$	C.C. ${}^{2}$	R.P. ${}^{3}$	B.R. ${}^{4}$	F.I.A.	C.C.	R.P.	B.R.
1	1312	115	26	17	1296	131	–
2	1311	24	49	18	1295	132	229
3	1310	24	–	19	1294	134	234
4	1309	119	77	20	1293	134	–
5	1308	119	–	21	1292	135	250
6	1307	121	111	22	1291	220	257
7	1306	121	–	23	1290	222	274
8	1305	123	128	24	1289	222	–
9	1304	123	–	25	1288	139	279
10	1303	208	155	26	1287	147	281
11	1302	33	181	27	1286	249	282
12	1301	33	–	28	1285	143	285
13	1300	211	211	29	1284	143	–
14	1299	129	217	30	1283	228	285
15	1298	129	–	31	1282	145	287
16	1297	38	223	32	1281	230	288

${}^{1}$ Fault Injection Attempt; ${}^{2}$ Clock Cycle of the attack; ${}^{3}$ Relative Position of the fault; ${}^{4}$ Number of bits Retrieved.

**Table 5:** Results obtained from the attack on Trivium 2.

F.I.A. ${}^{1}$	C.C. ${}^{2}$	R.P. ${}^{3}$	B.R. ${}^{4}$	F.I.A.	C.C.	R.P.	B.R.
1	1312	22	26	17	1296	38	–
2	1311	24	53	18	1295	132	273
3	1310	24	–	19	1294	134	278
4	1309	25	75	20	1293	134	–
5	1308	119	109	21	1292	43	279
6	1307	27	132	22	1291	43	–
7	1306	205	174	23	1290	45	280
8	1305	123	194	24	1289	45	–
9	1304	123	–	25	1288	47	283
10	1303	31	210	26	1287	47	–
11	1302	33	241	27	1286	49	284
12	1301	33	–	28	1285	50	284
13	1300	211	250	29	1284	50	–
14	1299	129	263	30	1283	228	284
15	1298	129	–	31	1282	53	287
16	1297	38	272	32	1281	55	288

${}^{1}$ Fault Injection Attempt; ${}^{2}$ Clock Cycle of the attack; ${}^{3}$ Relative Position of the fault; ${}^{4}$ Number of bits Retrieved.
